# The Long-Term Effectiveness of Interventions Addressing Mental Health Literacy and Stigma of Mental Illness in Children and Adolescents: Systematic Review and Meta-Analysis

**DOI:** 10.3389/ijph.2021.1604072

**Published:** 2021-12-15

**Authors:** Alexandra Maria Freţian, Patricia Graf, Sandra Kirchhoff, Gloria Glinphratum, Torsten M. Bollweg, Odile Sauzet, Ullrich Bauer

**Affiliations:** ^1^ Faculty of Educational Science, Bielefeld University, Bielefeld, Germany; ^2^ School of Public Health, Bielefeld University, Bielefeld, Germany

**Keywords:** adolescents, long-term effectiveness, mental health literacy, stigma, social distance, intervention, mental illness

## Abstract

**Objectives:** This study aims to provide a systematic review and meta-analysis of the literature on the long-term effects of interventions addressing children’s and adolescents’ mental health literacy and/or stigmatizing attitudes.

**Methods:** Articles in English or German published between January 1997 and May 2020 were retrieved from five databases, leading to a total of 4,375 original articles identified.

**Results:** 25 studies were included after applying exclusion criteria, 13 of which were eligible for meta-analysis. The overall average of the follow-up period was about 5 months. Long-term improvements were sustained for mental health literacy, d = 0.48, 95% CI = (0.34, 0.62), as well as for stigmatizing attitudes, d = 0.30, 95% CI = (0.24, 0.36), and social distance, d = 0.16, 95% CI = (0.03, 0.29). The combination of educational and contact components within interventions led to worse results for mental health literacy, but not stigmatizing attitudes or social distance.

**Conclusion:** Interventions targeting children and adolescents generally have a brief follow-up period of an average of 5 months. They show a stable improvement in mental health literacy, but are to a lesser degree able to destigmatize mental illness or improve social distance.

## Introduction

Most mental disorders emerge during childhood and adolescence. An estimated 75% of mental disorders have an onset before the age of 25, with 50% developing before the age of 14 [1]. Beyond the distress and impairment of mental disorders, they also have disruptive effects on academic achievement, personal relationships, and stability within the job market, bringing about negative social and financial consequences lasting into adulthood. Negative consequences do not only affect individuals during their experience of mental disorder, but can influence their mental health trajectory later in life [2, 3].

Globally, there exists a discrepancy between the need for mental health services and their (low) availability and utilization at all ages, with different impeding factors, e.g., limited resources or mental health knowledge [4]. In this paper, we focus particularly on two constructs considered as key factors for improving this situation: mental health literacy (MHL) and stigma.

*MHL* was first defined by Jorm et al. [5] as the “knowledge and beliefs about mental disorders which aid their recognition, management or prevention.” As such, it has been identified as a significant predictor for seeking help *via* mental health services [6]. More specifically, Tully et al. [7] suggest that an important factor contributing to inadequate utilization of mental health treatment options, particularly among younger people, may be low MHL levels among parents and community members. However, MHL is both a social and individual resource that can be fostered in young people. From a public health perspective, empowering children and adolescents to seek help for mental problems is crucial for secondary and tertiary prevention of mental illness (i.e., as cure or disorder management).

The stigma associated with mental illness also presents a significant barrier to help-seeking processes and has been described as “having worse consequences than the conditions themselves” [8]. This is partly because widespread prejudice and stereotypes (e.g., that those with mental illness are weak, dangerous, or inept) bring about discrimination on institutional and individual levels—for example, in the workplace, the health care system, or everyday social interactions [9]. Exposed to such stereotypes, people affected by mental illness may also stigmatize themselves, leading to low self-esteem, low self-efficacy, negative emotional reactions, and corresponding behaviors that further reduce quality of life [10]. In turn, self-stigma and/or fear of stigma can influence individuals to avoid seeking help in treatment form, with consequences for their further mental health [11].

As outlined, the prevalence of help-seeking behaviors when experiencing signs of mental illness is assumed to be deeply connected to perceived public perceptions of mental illness, including stigmatizing attitudes. Accordingly, stigma has frequently been examined in mental illness studies [12]. While many interventions for the improvement of mental health among children and adolescents exist, O’Reilly et al. [13] have called the lacking evidence base and the question of their long-term efficacy to attention. Moreover, while interventions tackling stigma have been reviewed more often [14–16], less knowledge is available for MHL. At the same time, to our knowledge, the existing reviews on interventions for MHL improvement in children and adolescents, do not tackle the question of long-term effectiveness. Important contributions in the MHL field, differing in aim and scope, were conducted by Wei et al. [17] (assessing general intervention effectiveness), Seekadet et al. [18] (assessing intervention types’ effectiveness), and Patafio et al. [19] (providing an overview of intervention programs). Some limitations of the Wei et al. [17] and Patafio et al. [19] studies were their inclusion of studies lacking control conditions, indicating weaker study designs and less accurate results. In contrast, this review only includes studies with control conditions and focuses specifically on long-term intervention effectiveness.

### Aim

We aim to provide a systematic review and meta-analysis of interventions that aim to improve young peoples’ MHL and/or to reduce mental illness related stigma in the target group. We investigate MHL and stigma as separate but possibly related outcomes of intervention programs, while acknowledging that—as currently understood—MHL is a multidimensional construct that may incorporate stigma among other aspects. However, we regard MHL and stigma as distinct outcomes to avoid the ambiguity and heterogeneity that arises when studies measuring distinct aspects are summarized under the overarching label “MHL” [20].

Additionally, to narrow the knowledge gap regarding long-term effects of intervention studies [14, 15], our focus lies on interventions that follow up on their results by incorporating three measurement time points.

Research question: Do mental health intervention programs addressing children and adolescents effectively 1) reduce stigma related to mental illness and/or 2) improve MHL long-term?

## Methods

### Search Methodology

To find evaluated interventions addressing mental health-related stigma and/or MHL in children and adolescents, the following databases were searched for articles in English or German published from 1997 onward—when the term “mental health literacy” was introduced by Jorm et al. [5]: PubMed, PsycINFO, PSYNDEX, ERIC, and Web of Science Core Collection. PubMed and Web of Science were searched directly; the others were accessed *via* the research platform EBSCOhost. One search phase was conducted by searching for articles published between 1997 and May 2018 by two of our researchers. Since we were unable to finalize the publication due to time constraints, we added another search phase between May 2018 and May 2020 to keep results current. During both search phases, the same search algorithm was used.

The search strategy was developed through an iterative process with team members. Feedback was also obtained from librarians and external experts. The search term was adapted for each database. See Supplementary Figure S1 of the supplementary material for an example of the search string used in PubMed.

Additionally, a manual search was conducted by contacting experts and organizations and manually searching through the references of key publications found through the database search.

### Inclusion and Exclusion Criteria

Studies were included if they:

1) addressed children and adolescents. Although we chose the legal age of consent of 18 years as an orientation point, we did not exclude studies with a few participants older than 18,
2) included three measurement points: one pre-intervention point, one post-intervention point and one follow-up assessment,
3) delivered an intervention program,
4) had a control group or provided an intervention as treatment as usual,
5) assessed the mental health-related stigma and/or MHL directly through the self-report of children or adolescents, instead of relying on information from caregivers or teachers.

Studies were excluded if they:

1) had no information about participants’ age or affiliation to the educational system (so it could not be inferred whether they were underaged),
2) did not directly measure the MHL and/or stigma for children and adolescents, but through representatives such as parents or teachers,
3) did not report results (e.g., abstracts of registered clinical trials).

### Selection Process

The identified citations, together with their bibliographic records, such as title, abstract, and keywords, were imported from the databases into a reference management program.

First, duplicate publications were automatically removed by the program and manually verified in two phases by two researchers. Second, 200 abstracts were jointly screened by the same researchers to establish the rate of agreement between them. The initial interrater reliability resulted in an overlap of 86.5% and after disagreements were discussed, it was optimized to 100% in a second joint screening.

Third, title/abstract screening was performed independently by the same two researchers in the first phase and independently by one of them in the second phase. One researcher screened the PsycINFO and Web of Science results, while another screened those from PubMed, ERIC, and PSYNDEX. In the second phase, all databases were searched by one researcher.

Fourth, the full texts of the included studies were obtained through the university library. If articles were inaccessible, their authors were contacted. Full-text screening was then conducted by the same two researchers together in the first phase and by one of them in the second phase. Uncertainties regarding study eligibility were resolved through discussion between the two researchers until a consensus on inclusion or exclusion was reached. Finally, information from the included articles was extracted into tables created for this review. These tables cover characteristics of the sample and characteristics of the intervention.

### Quality Assessment

Two authors rated the included studies’ quality using the *Checklist for quality assessment of controlled intervention studies*, a free resource from the National Heart, Blood and Lung Institute [21] comprising 14 questions. They rated each question and resolved discrepancy through discussion. The overall assessment was based on a point system factoring in the checklist’s guiding elements. One question regarding participant blinding was not included in calculation of the final score, as in most cases, blinding is not feasible when providing educational interventions. Moreover, we complemented the intervention fidelity assessment criteria with the criterion of a training provision for the facilitators of an intervention. This, however, was also omitted from the final score. One point was given for each question answered with “yes” except for question 13 regarding the prior specification of reported outcomes or subgroups. Here, we differentiated more distinctly between two possibilities. Studies with a preregistered study protocol were granted one point; studies mentioning the change of predefined outcomes as a study aim were granted 0.5 points. The possible final score could range from zero points, indicating a very high risk of bias, to 13 points, indicating a low risk of bias.

### Data Analysis

We computed weighted means for the follow-up lengths and participants’ ages. When the follow-up period was reported as a range, we used the mean of that range. We intended to calculate odds ratios for dichotomous outcomes, but this applied to only two studies. We verified which studies reported means and standard deviations for the main outcomes and included those into a meta-analysis using a random effects model. The analysis was conducted by one author using STATA 16 [22]. Due to the heterogeneity of measurement tools, the measured outcomes were regrouped into three categories [1]: MHL [2], stigmatizing attitudes, and [3] social distance. Higgins’s I^2^ test was used to estimate “the proportion of variation between the sample estimates that is due to heterogeneity rather than to sampling error” [23]. If I^2^ has a value of 50% or more it is considered to have significant heterogeneity [23].

The categories were built through inductive reasoning, considering both conceptualization and operationalization of the measures used. When articles did not explicitly mention the construct measured, the measurement instruments were carefully considered and categorized as follows:

• MHL: items regarding knowledge,
• stigma: items about attitudes (e.g., agreement regarding stereotypes about mental illness), and
• social distance: willingness to interact with a person with mental illness in varying contexts.

More detailed differentiation in terms of MHL (e.g., separating general MHL and more specific knowledge on particular disorders, e.g., depression literacy, etc.) and stigma (e.g., self-stigma) was considered, however, too few studies were available to support this categorization.

Moreover, we verified whether the length of post- or follow-up assessment affected the outcomes with linear meta-regression. We also assessed a possible effect of the intervention type (educational intervention vs. educational plus contact intervention) and study design [randomized controlled-trials (RCT) vs. non-RCT].

## Results

**FIGURE 1:**
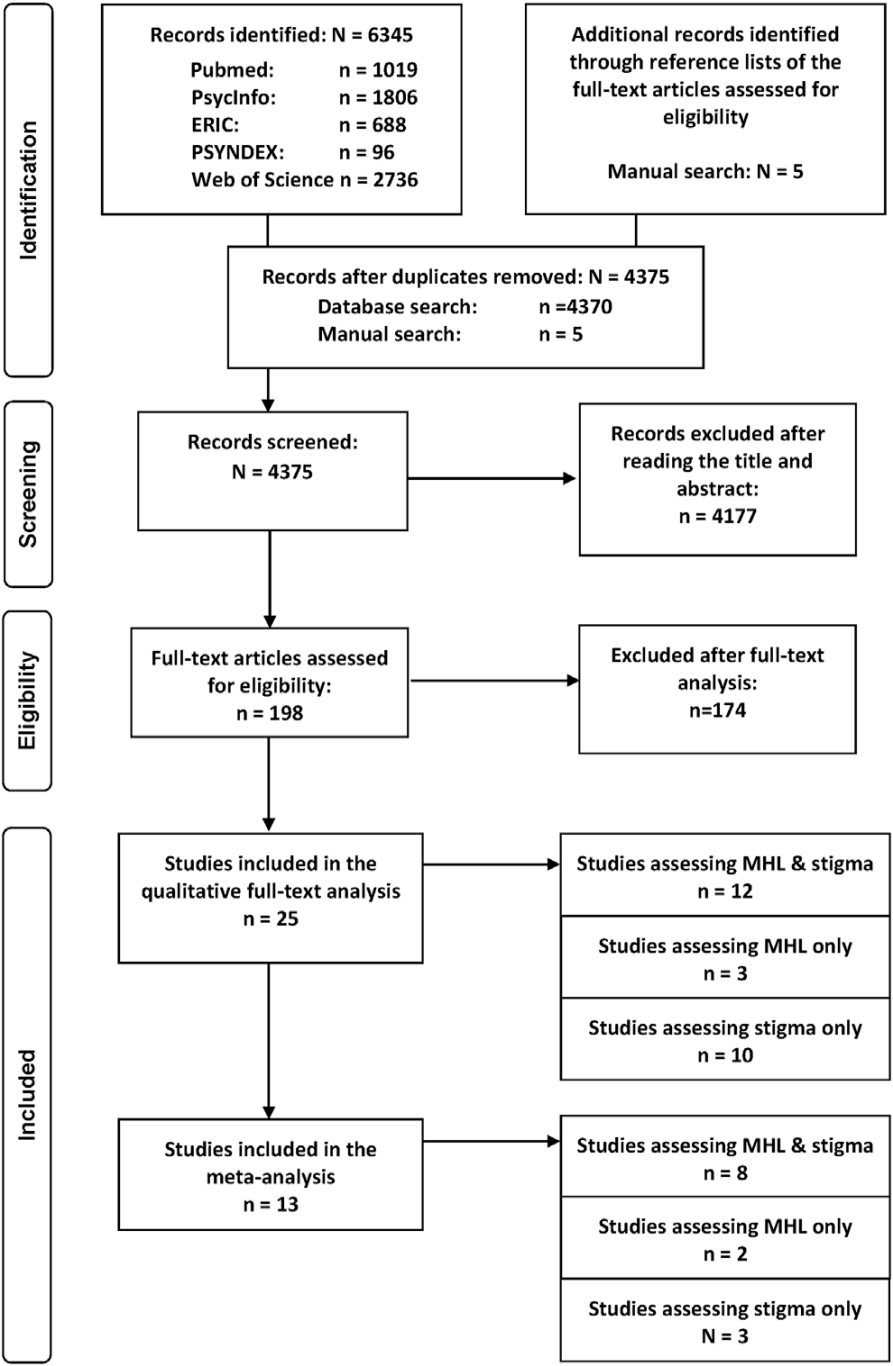
Prisma flow-chart of papers included in the review regarding the long-term effectiveness of interventions addressing mental health literacy and stigma of mental illness in children and adolescents, search period: January 1997 to May 2020. Project: Improving mental health literacy to reduce stigma (IMPRES), Bielefeld, Germany, 03.2018 - 02.2021.

The search provided 6,345 records, of which 1,975 were duplicates. The manual search revealed an additional 5 articles. The screening and selection process is illustrated in more detail in Figure 1. In total, 25 studies were included for the analysis. Ten studies measured stigma only, three studies MHL only, while twelve studies assessed both.

17 studies were retrieved during the first search period (up until May 2018) and 8 during the second search period (from May 2018 to May 2020). As defined in the inclusion/exclusion criteria, all of the studies employed three measurement time points. However, this only applied to the intervention group. Four studies used only two measurement points in the control group [numbers (nos.) 1, 5, and 11 did not have a follow-up measurement, while no. 6 did not measure the post-assessment, but directly the follow-up], but were not excluded in order to maintain a larger database. On average, the follow-up time was 5 months after intervention finalization. When studies reported more than two post-measurement time points, the first and the last post-test measurements were considered.

### Descriptive Summary of Included studies

A summary of key study characteristics is displayed in Table 1 and descriptions of each study can be found in Table 2.

**TABLE 1:** Studies assessing the long-term effects on MHL and/or stigma in children or adolescents: a summary of intervention/study characteristics, search period: January 1997 to May 2020. Project: Improving mental health literacy to reduce stigma (IMPRES), Bielefeld, Germany, 03.2018 - 02.2021.

	All studies included in the review (*n* = 25)	Only studies eligible for meta-analysis (*n* = 13)
Sample size	*n* = 18,157	*n* = 6528
Study design	52% used a randomized controlled design (at different levels: school, class, participant)	38.5% used a randomized controlled design (at different levels: school, class, participant)
Length of follow-up in weeks	m = 23.14 (weighted)	m = 23.73 (weighted)
	range = 4.3 to 103.2	range = 6 to 103.2
Participants’ age	m = 14.55 (weighted), range = 9–21	m = 14.50 (weighted), range = 9–18
Continent	• Europe: 9 studies	• Australia: 5 studies
	• North America: 6 studies	• North America: 4 studies
	• Australia: 7 studies	• Europe: 2 studies
	• Asia: 3 studies	• Asia: 2 studies
Setting	76% of studies were conducted in school, while others were conducted in the community, in school/sport clubs, or with clinical population	69.23% of studies were conducted in school, while others were conducted in the community, in school/sport clubs, or with clinical population
Type of administered intervention	• educational intervention: 11 studies	• educational intervention: 8 studies
	• educational and contact intervention: 10 studies (in person contact: 8 studies, in person + video contact: 1 study, video contact: 1 studies)	• educational and contact intervention: 3 studies (personal contact: 2 studies, in person + video contact: 1 study)
	• comparison between education and education + contact intervention: 1 study	• indirect intervention trough parental training: 1 study
	• contact intervention (video): 1 study	• unknown: 1 study
	• indirect intervention through parental training: 1 study	
	• unknown: 1 study	
General intervention topic	• general MH: 17 studies	• general MH: 9 studies
	• specific MH: 7 (depression 4, schizophrenia 3)	• specific MH: 3 (depression 3)
	• unknown: 1 study	• unknown: 1 study
Duration of intervention	range: <1–18 h	range: <1–18 h
	• up to 1 h: 5 studies	• up to 1 h: 1 study
	• > 1–5 h: 8 studies	• > 1–5 h: 5 studies
	• > 5–9 h: 3 studies	• > 5–9 h: 0 studies
	• > 9 h: 6 studies	• > 9 h: 4 studies
	• unknown: 3 studies	• unknown: 3 studies
Timespan of intervention	range: 1 day to 2,5 months	range: 1 day to 2,5 months
	• up to 1 day: 10 studies	• up to 1 day: 3 studies
	• > 1 day, < 1 week: 6 studies	• > 1 day, < 1 week: 5 studies
	• > a week, < a month: 2 studies	• > a week, < a month: 0 studies
	• > a month: 4 studies	• > a month: 3 studies
	• unknown: 3 studies	• unknown: 2 studies
Person who delivered intervention	• (MH) professional [5]/teacher [1]/researcher [1]/unknown [1] + person with personal experience: 8 studies	• (MH) professional [1]/teacher [1] + person with personal experience: 2 studies
	• teacher (+researcher [1]): 5 studies	• teacher (+researcher [1]): 4 studies
	• (MH) professional: 4 studies	• (MH) professional: 3 studies
	• trained presenter: 3 Studies	• trained presenter: 1 Studies
	• person with personal experience: 2 studies	• person with personal experience: 1 study
	• unknown: 4 studies (note: *n* = 26; one study used two different delivery persons to compare modes)	• unknown: 3 studies (note: *n* = 14; one study used two different delivery persons to compare modes)
Didactic materials	Multi-method: 21 studies; primarily one method: 2 studies; unknown: 2 studies	Multi-method: 10 studies; primarily one method: 1 study; unknown: 2 studies
	• informative/educational presentations: 21	• informative/educational presentations: 11
	• exercises/games/role-plays: 15 studies	• exercises/games/role-plays: 8 studies
	• guided discussions: 12	• guided discussions: 5
	• Q&A session regarding personal experience with MI: 9 studies	• Q&A session regarding personal experience with MI: 4 studies
	• informative video: 8	• informative video: 4

M, average; SD, standard deviation; MH, mental health; H, hour; Q&A, question and answer; MI, mental illness.

**TABLE 2:** Studies assessing the long-term effects on MHL and/or stigma in children or adolescents: a presentation of individual studies, search period: January 1997 to May 2020. Project: Improving mental health literacy to reduce stigma (IMPRES), Bielefeld, Germany, 03.2018 - 02.2021.

Study				Sample					Study design and outcomes				Intervention	
No.^a^	Authors	Country	Setting^b^	Participant age (mean)	Participant age (SD)	Participant age range^c^	Sample size^d^	Length follow-up in weeks^e^	Risk of bias^f^	Randomization	Assessed outcomes^g^	Outcome categorization^h^	Type of intervention^i^	Intervention topic^j^
1	Ahmad et al. (2019) [20]	United States	school clubs	not reported	not reported	not reported	545	15.05	3.5	yes, at school level	MHL, S	MHL, SA, SD	unknown	unknown
2	Andrés-Rodríguez et al. (2017) [21]	Spain	school	14.24	0.47	14–18	393	38.7	4.5	no	S	SA, SD	E + C	general MH
3	Campos et al. (2018) [22]	Portugal	school	13.04	0.79	12–14	543	25.8	2.5	yes, at class level	MHL	MHL	E	general MH
4	Esters et al. (1998) [23]	United States	school	14.7	not reported	13–17	40	12	2	no	S	SA	E	general MH
5	Fraser et al. (2008) [24]	Australia	clinical population (parents and 1/3 children with MI)	13.34	1.58	12–17	44	19	0.5	no	MHL	MHL	E	general MH
6	Ibrahim et al. (2020) [25]	Malaysia	clinical population (symptoms of MI)	14.61	1.39	13–17	101	12.9	4.5	no	MHL, S	MHL, SA	E	specific MH: depression
7	Lai et al. (2016) [26]	China	school	15.1	1	14–16	3391	19.35	4.5	no	MHL, S	MHL SA	E	specific MH: depression
8	Morgan et al. (2019) [27]	Australia	community	13.3	1.54	12–15	301	103.2	10	yes, at participant level	S	SA, SD	indirect: E for parents	general MH
9	Perry et al. (2014) [28]	Australia	school	14.8	0.73	13–16	380	25.8	7	yes, at class level	MHL, S	MHL, SA	E	general MH
10	Pinto-Foltz et al. (2011) [29]	United States	school	15	0.67	13–17	156	8	6.5	yes, at class level	MHL, S	MHL, SD	E + C	general MH
11	Robinson et al. (2010) [30]	Australia	school	15.2	0.5	14–16	246	10.6	1.5	no	MHL, S	MHL, SA, SD	E + C (C in person and via video)	specific MH: depression
12	Ventieri et al. (2011) [31]	Australia	school	10.67	0.89	9–12	195	17.2	1.5	no	MHL, S	MHL, SA, SD	E	general MH
13	Wahl et al. (2011) [32]	United States	school	12.5	0.6	7th, 8th grade	193	6	1.5	no	MHL, S	MHL, SA, SD	E	general MH
14	Campbell et al. (2010) [33]	United Kingdom	school	14.64	0.48	14–15	92	10	6.5	yes, at class level	S	SA	E + C	specific MH: psychosis (categorized as schizophrenia)
15	Chisholm et al. (2016) [34]	United Kingdom	school	12.21	0.58	12–13	769	25.8	9	yes, at class level	MHL, S	MHL, SD	E vs. E + C	general MH
16	Conrad et al. (2009) [35]	Germany	school	not reported	not reported	13–18	210	12.9	1.5	no	S	SD	E + C	general MH
17	Economou et al. (2011) [36]	Greece	school	13.84	0.82	13–15	616	51.6	5.5	yes, at class level	S	SA	E	specific MH: schizophrenia
18	Goncalves et al. (2015) [37]	Portugal	school	not reported	not reported	7th, 8th, 9th grade	207	4.3	3.5	yes, at class level	S	SA, self-stigma	C (via video)	general MH
19	Hart et al. (2019) [38]	Australia	school	15.87	0.52	15–17	1605	51.6	7	yes, at school level	MHL	MHL	E	general MH
20	Liddle et al. (2021) [39]	Australia	football club	14.3	1.75	12–18	102	4.3	9	yes, at team/team-age level	MHL, S	MHL, SA	E + C	general MH
21	Mulfinger et al. (2018) [40]	Germany	clinical population (diagnosed with MI)	15.75	1.63	not reported	98	6	9	yes, at participant level within clusters	S	self-stigma	E + C	general MH
22	Ng et al. (2002) [41]	China	school	15	not reported	13–21	169	30.1	3.5	no	S	SA	E + C	general MH
23	Schulze et al. (2003) [42]	Germany	school	15.1	not reported	14–18	150	4.3	1.5	no	S	SA, SD	E + C	specific MH: schizophrenia
24	Swartz et al. (2017) [43]	United States	school	not reported	not reported	14–15	6679	17.2	3	yes, at school level	MHL, S	MHL, SD	E	specific MH: depression
25	Wahl et al. (2018) [44]	United States	school	14.7	not reported	13–18	932	5	1.5	no	MHL, S	MHL	E + C (C via video)	general MH

a Studies no. 1 to 13 were included in the meta-analysis.

b MI, mental illness.

c where not available, school grade is reported.

d at baseline of data collection.

e where months were reported, the number was multiplied by 4.3; where only a timespan was mentioned, the average was used; if there were two follow-up timepoints the last one was reported.

f Range: 0 to 13, higher scores indicating less risk of bias.

g MHL, mental health literacy; S, stigma.

h MHL, mental health literacy; SA, stigmatizing attitudes; SD, social distance.

i E, education, C, contact.

j MH, mental health.

The studies were conducted in nine different countries. All but six were conducted in schools, usually during regular lessons (other settings: clinical context: *n* = 3; football/school club: *n* = 2; community: *n* = 1). Most studies included both males and females, however two (nos. 11 and 20) targeted males only, while one targeted females only (no. 10). The average age was 14.55 years and ranged from nine to 21 years. A detailed description of the quality assessment of the included studies can be found in Supplementary Table S1 of the supplementary material. The average quality score was 4,4 (SD = 2,9).

Overall, two intervention types were predominant: educational interventions (*n* = 11) and educational plus contact interventions (*n* = 10). The remaining four studies had differing approaches: one compared educational and educational plus contact interventions (no. 15), one only contained contact with a person who experienced mental illness *via* video (no. 18), and one indirect intervention addressed parents (no. 8). Finally, in one study, the intervention type was not reported clearly (no. 1). Most interventions addressed general mental health topics (*n* = 17). Some targeted specific mental health issues, focusing on either depression (*n* = 4) or schizophrenia (*n* = 3).

Generally, interventions diverged in content and organization. Their durations ranged from under 1 h to a maximum of 18 h. Over half (13 out of 22) had a rather short duration of up to 5 h, while three studies did not report the intervention duration.

Considering the interventions’ timespans, most interventions (*n* = 10) can be described as short-term, i.e., delivered within 1 day. Eight were categorized as mid-term, lasting up to 1 week (*n* = 6) or up to 1 month (*n* = 2). The remaining four “long-term” interventions had a length of over 1 month.

A team of teachers and mental health professionals were frequently responsible for administering the interventions. One study compared effectiveness of delivery by mental health professionals to delivery by teachers (no. 7). Also, different modalities were used for delivering intervention content, including educational presentations and/or videos combined with various interactive parts (e.g., exercises, games, role-plays, guided discussions, etc). Some studies (*n* = 9) also included personal contact, where a person with mental illness experience shared their knowledge and responded to students’ questions.

### Effectiveness of Interventions in Reducing Stigma and Improving MHL

Means and standard deviations were retrieved from studies where they were reported (*n* = 12). One author provided this data upon request (no. 13), however, we were unable to obtain additional information from the other studies. One study (no. 15) reported all necessary means, but did not have a control group, rather comparing two different versions of the same intervention. This study was excluded from meta-analysis since it would have skewed effectiveness.

One of the studies (no. 7) compared two different delivery options of the same intervention (i.e., implemented by professionals vs. by teachers) and included a control group. We divided this study into two separate entries, using the same control group as a reference. Therefore, when reporting the number of participants on which each outcome was based, we subsequently subtracted the equivalent of the additional control group. The additional control group is included in the figures on the meta-analysis.

Thirteen independent studies in total were included in the meta-analysis. Five used randomizations (nos. 1, 3, 8, 9, 10), while the others employed a convenience sample. The weighted average of the follow-up time was almost 24 weeks (range: 6 weeks to 2 years).

Across all studies, the regression analysis indicated that time had no significant effect on the stability of the follow-up results, meaning that retention of learned knowledge and improvement in attitudes (including the desire for social distance) seemed stable over the measured time period.

The non-significant results of the meta-regression indicated that the intervention type (contact plus education, education, and unspecified) neither affected stigmatizing attitudes nor the desire for social distance at post- and follow-up assessment. The MHL outcomes, however, were significantly worse for the contact-based intervention compared to the educational intervention at post-assessment [*β* = −0.84; 95% CI = (−1.51, −0.16); SE = 0.35; *p* < 0.05] and at follow-up [*β* = −0.46, 95% CI = (− 0.87, −0.04); SE = 0.21, *p* < 0.05]. The unknown study type also showed significantly worse outcomes at post-assessment [*β* = −1.02; 95% CI = (−1.66, −0.38); SE = 0.33; *p* < 0.01].

At post-measurement, RCTs obtained significantly lower scores than other study designs for stigmatizing attitudes [*β* = −0.27; 95% CI = (−0.52, −0.02); SE = 0.13; *p* < 0.05], social distance [*β* = −0.26, 95% CI = (−0.49, −0.04), SE = 0.11, *p* < 0.05] and MHL [*β* = −0.67, 95% CI = (−1.08, −0.26), SE = 0.21, *p* = 0.001]. At follow-up, RCTs had significantly lower results only for MHL [*β* = −0.39, 95% CI = (−0.66, −0.13), SE = 0.13, *p* < 0.01], while no significant effect of study design could be observed for stigmatizing attitudes and social distance.

#### Mental Health Literacy

**FIGURE 2:**
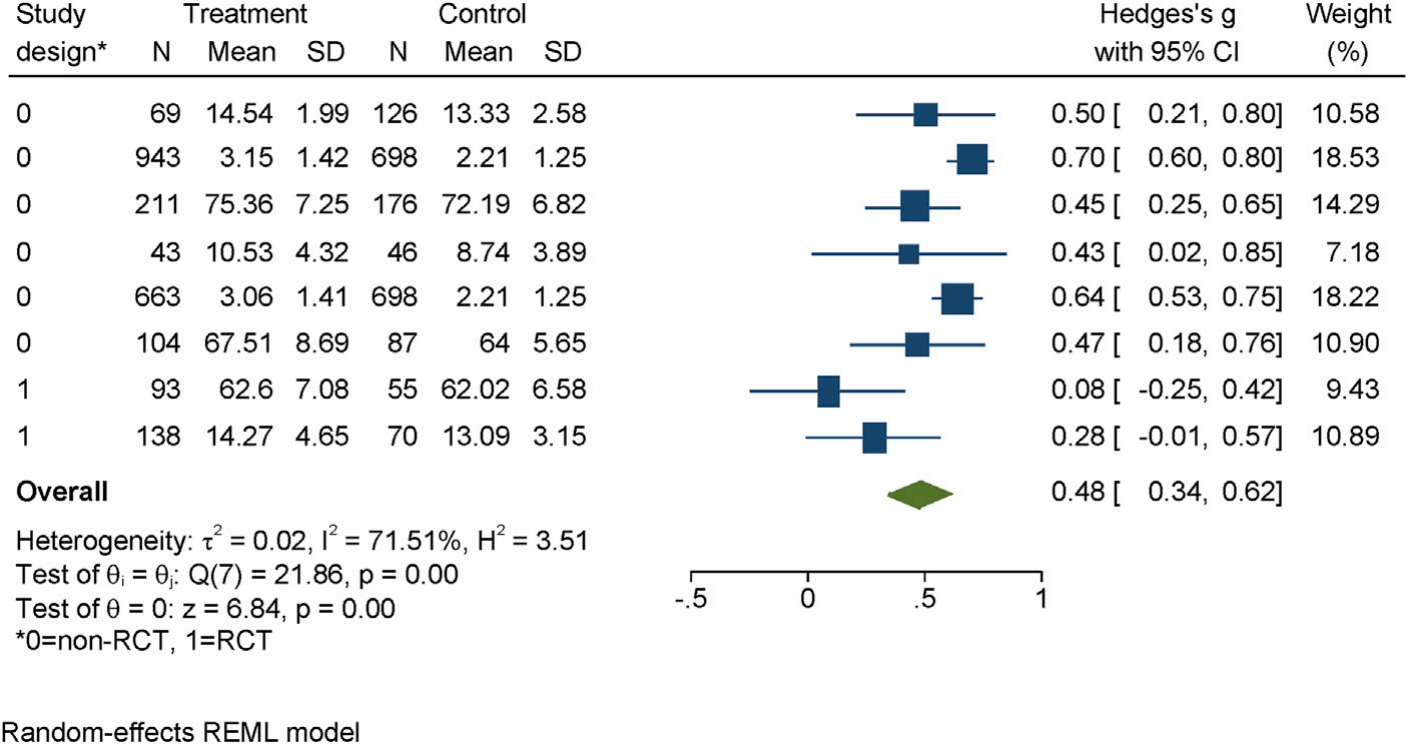
Evidence for effectiveness of interventions to improve MHL at follow-up assessment identified within the review regarding the effectiveness of long-term interventions addressing mental health literacy and stigma of mental illness in children and adolescents, search period: January 1997 to May 2020. Project: Improving mental health literacy to reduce stigma (IMPRES), Bielefeld, Germany, 03.2018 - 02.2021.

We were able to use data from eight studies, including 3,979 participants, to assess the immediate efficacy of interventions on MHL, as well as seven studies, including 3,522 participants, for the long-term follow-up (see Figure 2). The interventions could significantly improve MHL immediately after the intervention (one to 2 weeks afterwards) [d = 0.62, 95% CI = (0.34, 0.91)]. These effects diminished slightly over time (average of 23.62 weeks, range 6–25.8 weeks), but remained significant with a medium effect size [d = 0.48, 95% CI = (0.34, 0.62)] (see Figure 2). At both times, high heterogeneity was observed across studies: 94.44% at post-assessment and 71.51% at follow-up. Two studies showed negative, but not significant results at post- and follow-up assessment.

#### Stigmatizing Attitudes

**FIGURE 3:**
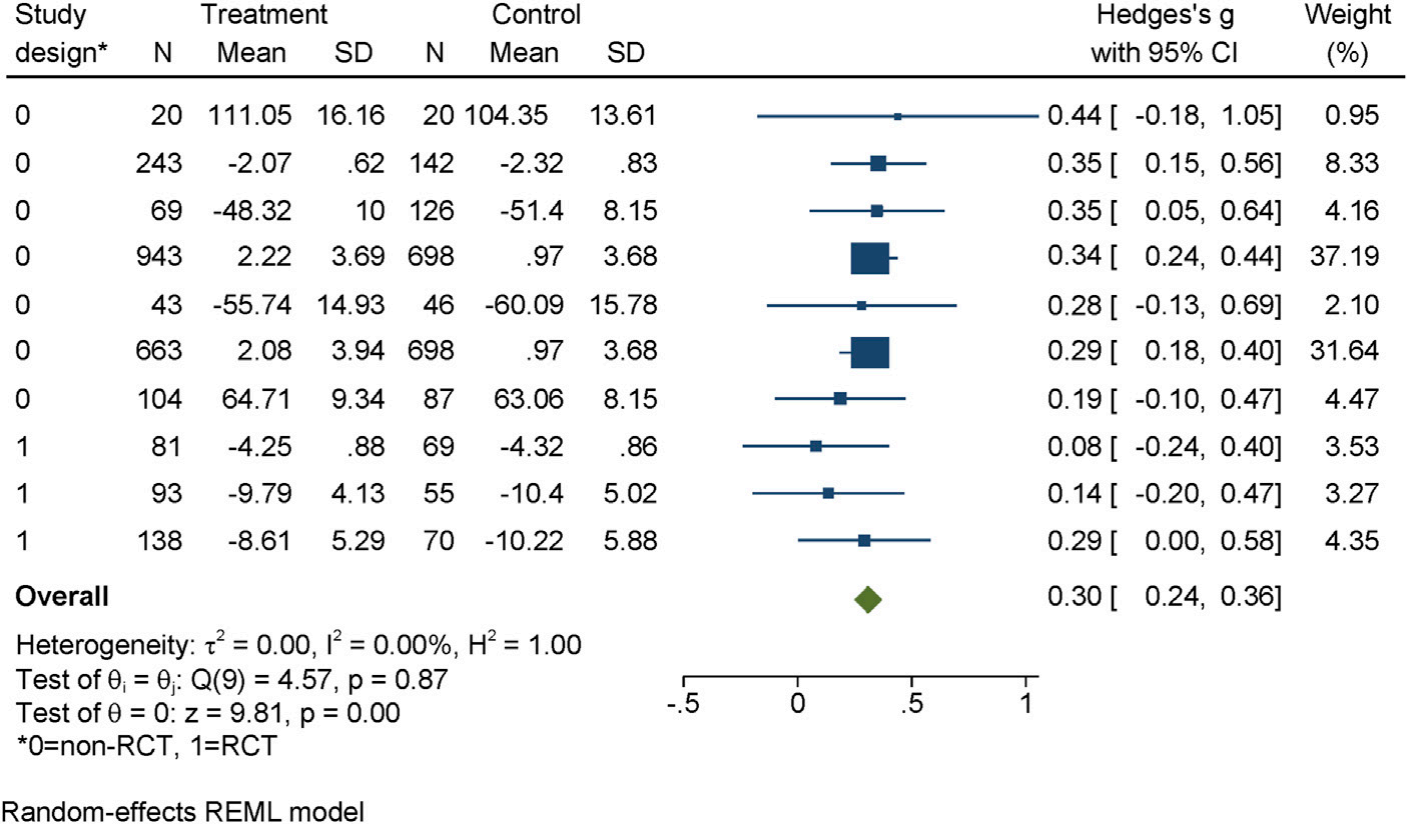
Evidence for effectiveness of interventions to reduce stigmatizing attitudes at follow-up assessment identified within the review regarding the effectiveness of long-term interventions addressing mental health literacy and stigma of mental illness in children and adolescents, search period: January 1997 to May 2020. Project: Improving mental health literacy to reduce stigma (IMPRES), Bielefeld, Germany, 03.2018 - 02.2021.

Stigmatizing attitudes were measured by ten studies (including 4,272 participants) at post-assessment and nine studies (including 3,710 participants) at follow-up. The post-measurement was homogenous (one to 2 weeks after intervention) for all but one study, which first assessed the program after 52 weeks post-intervention. The average follow-up period was 23.62 weeks (range between 6 and 103 weeks). The interventions showed stable improvement over time, with effect sizes of d = 0.30, 95% CI = (0.17, 0.43) at post-assessment and d = 0.30, 95% CI = (0.24; 0.36) at follow-up (see Figure 3). At post-test, three studies, all RCTs, showed non-significant improvements of stigmatizing attitudes, while the others were significant. At follow-up, two-thirds of the studies showed positive, non-significant improvements. Only two of these, however, were RCTs. Moreover, one single study split into two conditions accounts for considerably more weight (68.83%) of the results in the follow-up. While the heterogeneity was high at post-assessment (I^2^ = 74.13%), at follow-up, it was 0.00%.

#### Social Distance

**FIGURE 4:**
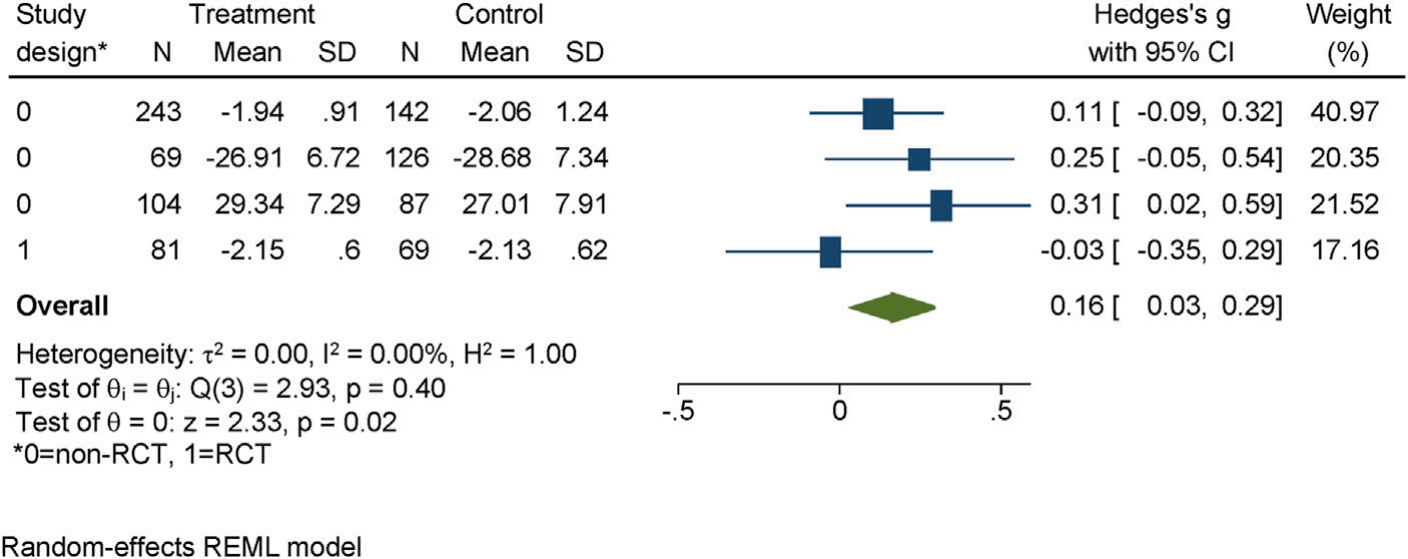
Evidence for effectiveness of interventions to reduce social distance at follow-up identified within the review regarding the effectiveness of long-term interventions addressing mental health literacy and stigma of mental illness in children and adolescents, search period: January 1997 to May 2020. Project: Improving mental health literacy to reduce stigma (IMPRES), Bielefeld, Germany, 03.2018 - 02.2021.

Six studies assessed social distance at post-assessment with 2908 participants and four at follow-up with 921 participants. The post-measurement was homogenous (usually 1 week after intervention) for all but one study, which first assessed the program after 52 weeks post-intervention. The average time until follow-up was almost 38 weeks (range from 6 to 103 weeks). Slight improvements were observed at post-intervention [d = 0.14, 95% CI = (0.02; 0.25)], and at follow-up [d = 0.16, 95% CI = (0.03, 0.29)] (see Figure 4). The heterogeneity at post-test was 17.74% and zero at follow-up. The non-significant studies outnumbered the significant ones: four out of six at post-test and three out of four at follow-up.

## Discussion

To our knowledge, this is the first study considering interventions aiming at the long-term reduction of stigma or improvement of MHL in children or adolescents. We found 25 controlled follow-up studies addressing either MHL, stigma, or both. Interventions typically took place in a school setting and were mostly implemented in the span of up to 1 week, with up to 9 h.

In some cases, content was delivered by staff external to schools (e.g., mental health professionals, researchers, etc.) and by teachers themselves in others. One comparative study found that knowledge and attitudes improved when both professionals and teachers delivered the intervention [30]. Matching previous recommendations [45–49], interventions involving school staff within a school setting could still be a convenient and effective option.

It is also relevant to consider the complexity of an intervention’s content. We found that most studies tackled mental health and mental illness as a general topic, while others focused on particularly common mental illnesses (e.g., depression) [50], or particularly stigmatized illnesses (e.g., schizophrenia) [51]. While learning about specific aspects regarding incidence, symptoms, and treatments of a particular illness is relevant, we argue that tapping into more general aspects, such as the stigma surrounding mental illness and strategies related to resilience and positive mental health, is just as relevant. To our knowledge, however, no study exists comparing the effectiveness of interventions addressing general mental health with those addressing specific mental illnesses.

Overall results indicate a positive stable improvement of MHL and, to a smaller degree, stigmatizing attitudes and social distance. As was the case in a review targeting long-term results in adults, we found that effect sizes for knowledge retention were higher than for attitudinal change [52]. The evidence is less clear for long-term effectiveness regarding stigma and social distance, since most of the included studies showed non-significant improvements in these areas. More research is needed in this respect, especially in identifying which conditions (e.g., content, person who delivers intervention, intervention setting, duration, etc.) lead to better outcomes. Put differently, although the evidence indicates overall that exposure to or engagement with information about mental health leads to improved MHL and slightly more positive attitudes related to mental illness, further research should go beyond asking whether interventions are effective and instead ask which components make some more effective than others.

In this respect, we found that educational interventions contributed to significantly improved knowledge retention over educational plus contact interventions. One randomized trial identified within this review, which was excluded from the meta-analysis due to lack of a control group, compared an educational intervention to an educational plus contact intervention. The content of both education parts was the same except for a short input on the history of mental illness, which replaced the personal contact. Despite the high content overlap, the education condition proved more effective than the education plus contact condition in improving MHL (recognizing a mental illness based on a vignette) and knowledge, but not stigmatizing attitudes. However, the education plus contact condition did not lead to significant improvement of stigmatizing attitudes over the education condition [38]. Our meta-analysis showed the same trend: results observed for stigma variables in the education condition were not significantly worse than the education plus contact condition. This is somewhat surprising compared to previous findings, since one review has shown that for adolescents (differently than for adults), educational interventions are more effective in reducing stigma than contact interventions [16]. Comparative studies, such as that conducted by Chisholm et al. [38] are necessary to identify which intervention components improve which particular areas of MHL.

Regarding outcomes, the mean duration between intervention and follow-up was almost five and a half months across all studies included in the meta-analysis, ranging from 6 weeks to 2 years. Surprisingly, longer durations until follow-up were unrelated to worse results, indicating stability of knowledge retention and attitudinal change. Thus, we can assume that the results might be stable across the studies’ identified ranges up to a maximum of 2 years. More research is needed to verify what happens beyond this time frame and whether repeated future interventions are needed to maintain positive change.

### Limitations

In the second search phase, only one of the researchers assessed study eligibility, which could lead to slightly biased results. Since a high agreement rate was reached within the first phase, however, we consider the risk of bias to be low.

Most identified studies reported positive significant results. This could be an indication of publication bias, especially related to the “file drawer problem” and thus overestimation of the identified effects. It has been estimated that publication bias can overestimate the treatment effect by up to 12% [53]. However, since our analytic method (random effect models) provides a more conservative estimate of the combined data [54], the bias in the obtained results might be limited.

Despite our focus on long-term follow-ups, outcome measurements took place, on average, 5 months after the intervention, making estimation of what happens beyond this time frame impossible. Thus, it is necessary to determine how long results are sustained by using longer follow-up periods, and whether one implemented intervention is sufficient or if the aim should be to repeat interventions regularly.

Another limiting aspect involves outcome categorization. The studies’ usages of different measurement tools for the same constructs renders them not readily comparable. We tried carefully assigning each outcome to the most appropriate category to reduce heterogeneity stemming from differences in outcome operationalization. Still, the MHL meta-analysis revealed high heterogeneity, both at post and follow-up assessment, while high heterogeneity across studies investigating stigma was only present at post-test. This is partially explainable by the regression analysis: the MHL results were influenced by study designs (RCT vs. non-RCT) and intervention types at both time points, while for stigma, the results were only influenced by the study design and not intervention type at only one of the measurement time points. High heterogeneity might be connected to other factors, such as content variability and intervention duration, measurement tool variability, and the target population. Overall, generalizability of the results must be considered critically and intervention program application should, preferably, be accompanied by evaluations.

Additionally, the overall risk of bias assessment indicated that most studies show high or moderate risks, while few have a low risk. The rather unfavorable assessment is partly based on the fact that studies do not report on all assessed aspects. We recommend that intervention studies follow reporting guidelines to overcome this information gap and offer more reliable results. In terms of randomization, as one of the used quality criterion, the great majority (11 of 13 studies) were randomized at a group level, most of which were school classes, while the rest were randomized at an individual level. Cluster randomized studies, where randomization takes place at a group level, require a larger sample size for reaching acceptable power to reveal significant results [55]. Thus, due to the inclusion of cluster-randomized studies, the results may be underestimated. Moreover, differences in results can occur due to the type of cluster chosen. Randomization at school level may also lead to underestimation of the results due to possible contamination bias, when pupils from different classes exchange information received within the intervention.

### Conclusion

We found 25 studies on interventions addressing MHL or stigma with varying content, delivery, and follow-up lengths. Schools were the predominant setting of delivery, where topics addressed were general mental health, depression, and schizophrenia. The meta-analysis indicates that interventions appear successful in improving MHL in the long term but provide less robust information on improving attitudes. We found that stigma and social distance did not vary across different intervention types, however, the education condition led to better MHL outcomes than the education plus contact condition. More studies are needed to identify which information should be conveyed in what way in order to successfully address both MHL and different aspects of stigma.
