# Supplementary material for: The Long-Term Effectiveness of Interventions Addressing Mental Health Literacy and Stigma of Mental Illness in Children and Adolescents: Systematic Review and Meta-Analysis
Source: Int J Public Health. 2021 Dec 15;66:1604072. doi: 10.3389/ijph.2021.1604072 (PMC8714636; doi:10.3389/ijph.2021.1604072)
Supplement: Supplementary file 1 [file Table1.DOCX]

Table 1 of the Supplementary material. Quality assessment of the included studies based on the National Heart, Blood and Lung Institute’s *Checklist for quality assessment of controlled intervention studies (CQACIS)*.

| **No.** | **Authors** | **1^[[1]](#footnote-1)^** | **2** | **3** | **4** | **5** | **6** | **7** | **8** | **9** | **9a*^[[2]](#footnote-2)^** | **10** | **11** | **12** | **13**** | **14** | **Total score** |
| --- | --- | --- | --- | --- | --- | --- | --- | --- | --- | --- | --- | --- | --- | --- | --- | --- | --- |
| 1 | Ahmad et al. (2019) (20) | yes | NA | NA | NA | NA | yes | no | NA | no | no | NA | yes | no | 2 | NA | 3.5 |
| 2 | Andrés-Rodríguez et al. (2017) (21) | no | NA | NA | yes | NA | yes | yes | yes | no | no | NA | yes | no | 2 | NA | 4.5 |
| 3 | Campos et al. (2018) (22) | yes | NA | NA | NA | NA | yes | no | no | no | 2 | NA | NA | no | 2 | NA | 2.5 |
| 4 | Esters et al. (1998) (23) | no | NA | NA | NA | NA | NA | yes | yes | NA | no | NA | NA | no | no | NA | 2 |
| 5 | Fraser et al. (2008) (24) | no | NA | NA | NA | NA | NA | no | no | no | 2 | NA | NA | no | 2 | NA | .5 |
| 6 | Ibrahim et al. (2020) (25) | no | NA | NA | NA | NA | yes | yes | yes | no | 2 | NA | yes | no | 2 | NA | 4.5 |
| 7 | Lai et al. (2016) (26) | no | NA | NA | NA | NA | no | yes | yes | yes | 1&2 | NA | NA | yes | 2 | NA | 4.5 |
| 8 | Morgan et al. (2019) (27) | yes | yes | yes | NA | yes | yes | no | yes | yes | 1 | NA | yes | no | 1 | yes | 10 |
| 9 | Perry et al. (2014) (28) | yes | yes | yes | NA | yes | no | no | no | no | 1 | no | yes | no | 1 | yes | 7 |
| 10 | Pinto-Foltz et al. (2011) (29) | yes | no | no | NA | NA | yes | yes | yes | yes | 1 | NA | no | no | 2 | yes | 6.5 |
| 11 | Robinson et al. (2010) (30) | no | no | no | NA | NA | NA | NA | NA | NA | no | NA | yes | no | 2 | NA | 1.5 |
| 12 | Ventieri et al. (2011) (31) | no | no | no | NA | NA | no | NA | NA | NA | no | NA | yes | no | 2 | NA | 1.5 |
| 13 | Wahl et al. (2011) (32) | no | no | no | NA | NA | yes | NA | NA | NA | no | NA | no | no | 2 | NA | 1.5 |
| 14 | Campbell et al. (2010) (33) | yes | no | no | NA | NA | NA | yes | yes | yes | 2 | NA | yes | yes | 2 | no | 6.5 |
| 15 | Chisholm et al. (2016) (34) | yes | yes | yes | NA | yes | NA | yes | yes | yes | 1 | NA | yes | no | 1 | yes | 9 |
| 16 | Conrad et al. (2009) (35) | no | no | no | NA | no | NA | NA | NA | NA | 1 | NA | yes | no | 2 | NA | 1.5 |
| 17 | Economou et al. (2011) (36) | yes | no | no | NA | no | yes | yes | yes | NA | NA | NA | yes | no | 2 | no | 5.5 |
| 18 | Goncalves et al. (2015) (37) | yes | no | no | NA | NA | no | NA | NA | yes | NA | NA | yes | no | 2 | no | 3.5 |
| 19 | Hart et al. (2019) (38) | yes | NA | yes | NA | NA | no | no | NA | no | 1 | yes | yes | yes | 1 | yes | 7 |
| 20 | Liddle et al. (2021) (39) | yes | yes | yes | yes | yes | NA | yes | yes | NA | 1 | NA | yes | no | 1 | yes | 9 |
| 21 | Mulfinger et al. (2018) (40) | yes | NA | no | NA | no | yes | yes | yes | yes | 1 | NA | yes | yes | 1 | yes | 9 |
| 22 | Ng et al. (2002) (41) | no | no | no | NA | no | yes | no | yes | NA | NA | NA | yes | no | 2 | NA | 3.5 |
| 23 | Schulze et al. (2003) (42) | no | no | no | NA | no | NA | NA | NA | NA | NA | NA | yes | no | 2 | NA | 1.5 |
| 24 | Swartz et al. (2017) (43) | yes | NA | NA | NA | NA | yes | no | no | NA | 1 | NA | NA | no | 1 | no | 3 |
| 25 | Wahl et al. (2018) (44) | no | no | no | NA | no | NA | no | no | yes | 1 | NA | no | no | 2 | NA | 1.5 |
| *1 = training, 2 = specialized staff;  **1 = having a study protocol, 2 = defining the modification of the outcomes as an aim | | | | | | | | | | | | | | | | | |

NA = not applicable/not reported

1. the criteria for each question are formulated in the CQACIS as follows:

   1. Was the study described as randomized, a randomized trial, a randomized clinical trial, or an RCT?

   2. Was the method of randomization adequate (i.e., use of randomly generated assignment)?

   3. Was the treatment allocation concealed (so that assignments could not be predicted)?

   4. Were study participants and providers blinded to treatment group assignment?

   5. Were the people assessing the outcomes blinded to the participants' group assignments?

   6. Were the groups similar at baseline on important characteristics that could affect outcomes (e.g., demographics, risk factors, co-morbid conditions)?

   7. Was the overall drop-out rate from the study at endpoint 20% or lower of the number allocated to treatment?

   8. Was the differential drop-out rate (between treatment groups) at endpoint 15 percentage points or lower?

   9. Was there high adherence to the intervention protocols for each treatment group? 10. Were other interventions avoided or similar in the groups (e.g., similar background treatments)?

   11. Were outcomes assessed using valid and reliable measures, implemented consistently across all study participants?

   12. Did the authors report that the sample size was sufficiently large to be able to detect a difference in the main outcome between groups with at least 80% power?

   13. Were outcomes reported or subgroups analyzed prespecified (i.e., identified before analyses were conducted)?

   14. Were all randomized participants analyzed in the group to which they were originally assigned, i.e., did they use an intention-to-treat analysis? [↑](#footnote-ref-1)
2. question 9a is not part of the CQACIS, but was introduced by us as an additional measure for the fidelity of the intervention, namely the standardization of intervention delivery trough training or trough specialized staff [↑](#footnote-ref-2)
