# Supplementary figures and images for: The Long-Term Effectiveness of Interventions Addressing Mental Health Literacy and Stigma of Mental Illness in Children and Adolescents: Systematic Review and Meta-Analysis
Source: Int J Public Health. 2021 Dec 15;66:1604072. doi: 10.3389/ijph.2021.1604072 (PMC8714636; doi:10.3389/ijph.2021.1604072)

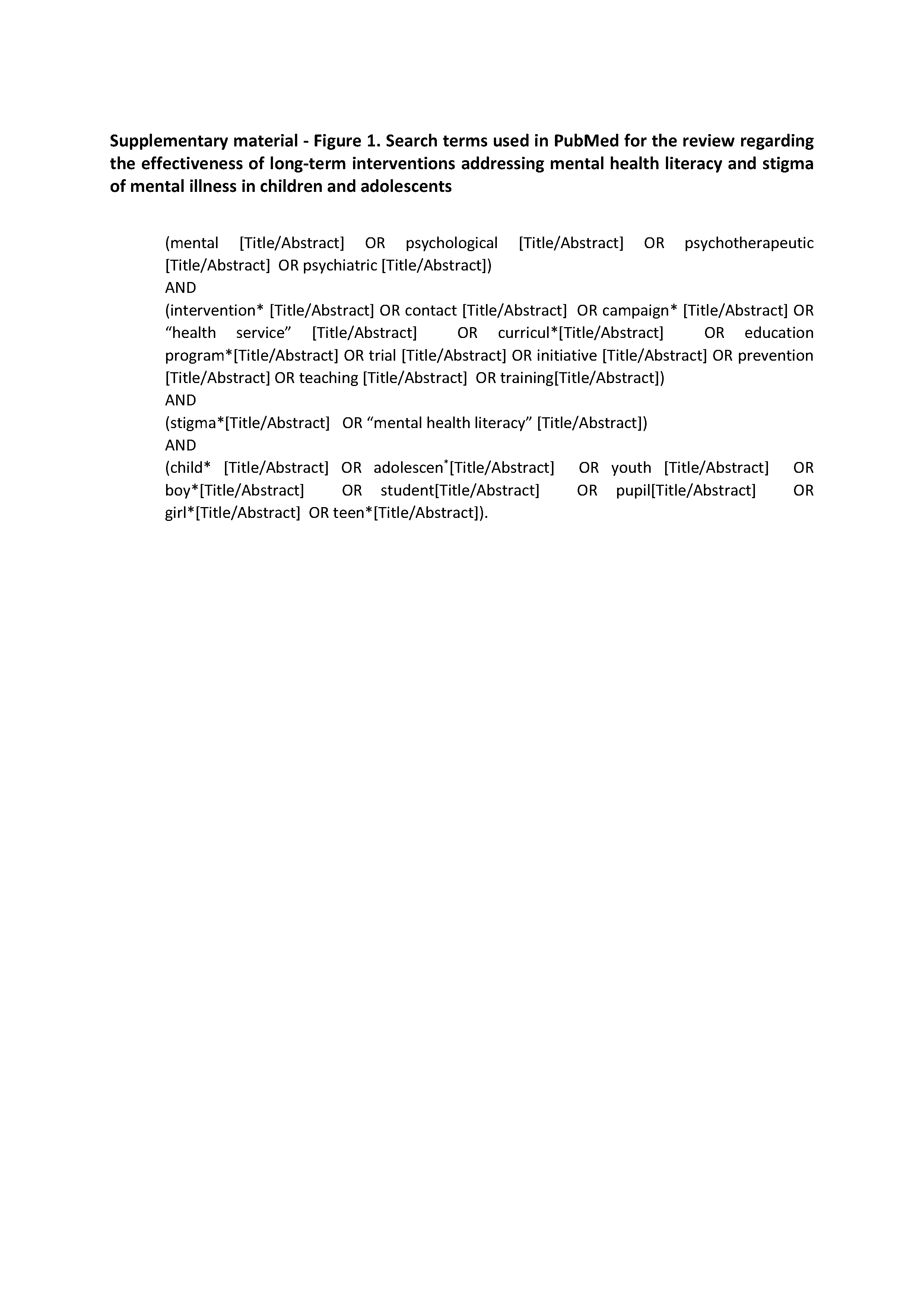

Supplement: Supplementary file 2 [file Image1.TIF]
